# Contrast enhancement on 100- and 120 kVp hepatic CT scans at thin adults in a retrospective cohort study: Bayesian inference of the optimal enhancement probability

**DOI:** 10.1097/MD.0000000000017902

**Published:** 2019-11-22

**Authors:** Takanori Masuda, Takeshi Nakaura, Yoshinori Funama, Tomoyasu Sato, Toru Higaki, Yoriaki Matsumoto, Yukari Yamashita, Naoyuki Imada, Masao Kiguchi, Yasutaka Baba, Yasuyuki Yamashita, Kazuo Awai

**Affiliations:** a Department of Radiological Technology, Tsuchiya General Hospital, 3-30 Nakajima-cho, Naka-ku, Hiroshima; b Department of Diagnostic Radiology, Graduate School of Medical Sciences; c Department of Medical Physics, Faculty of Life Sciences, Kumamoto University, 1-1-1 Honjo, Kumamoto; d Department of Diagnostic Radiology, Tsuchiya General Hospital, Nakajima-cho 3-30, Naka-ku; e Department of Diagnostic Radiology, Graduate School of Biomedical Sciences, Hiroshima University, Hiroshima, Japan.

**Keywords:** Bayesian analysis, contrast enhancement CT, hepatic CT, MDCT

## Abstract

**Purpose:**

To assess the probability of achieving optimal contrast enhancement in 100 kVp and 120 kVp-protocol on hepatic computed tomography (CT) scans.

**Materials and methods:**

We enrolled 200 patients in a retrospective cohort study. Hundred patients were scanned with 120 kVp setting, and other 100 patients were scanned with 100 kVp setting. We measured the CT number in the abdominal aorta and hepatic parenchyma on unenhanced scans and hepatic arterial phase (HAP)-, and portal venous phase (PVP). The aortic enhancement at HAP and the hepatic parenchymal enhancement at PVP were compared between the two scanning protocols. Bayesian inference was used to assess the probability of achieving optimal contrast enhancement in each protocol.

**Results:**

The Bayesian analysis indicated that when 100 kVp-rotocol was used, the probability of achieving optimal aortic enhancement (>280 HU) was 98.8% ± 0.6%, whereas it was 88.7% ± 2.5% when 120 kVp-protocol was used. Also, the probability of achieving optimal hepatic parenchymal enhancement (>50 HU) was 95.3% ± 1.5%, whereas it was 64.7% ± 3.8% when 120 kVp-protocol was used.

**Conclusion:**

Bayesian inference suggested that the post-test probability of optimal contrast enhancement at hepatic dynamic CT was lower under the 120 kVp than the 100 kVp-protocol

## 1 Introduction

Contrast material–enhanced computed tomography (CT) is the most commonly used imaging modality for the detection of hypervascular hepatic tumors such as hepatocellular carcinoma.^[1–5]^ For this application, it is crucial to achieve maximum hepatic enhancement to improve tumor-to-liver contrast on images. In order to improve the detection of hepatic tumors at CT, it is essential to achieve sufficient contrast enhancement of at least 280 hounsfield unit (HU) with hepatic arteries^[6]^ and 50 HU with hepatic parenchyma.^[7]^ Yamashita et al,^[8]^ reported that the dose of intravenous contrast material should be adjusted to the patient's weight to achieve adequate contrast enhancement and recommended the use of 2.0 to 2.5 mL of contrast material per kilogram of body weight, with an iodine concentration of 300 mgI/ml.

However, these reports evaluated the average enhancement of the abdominal aorta or the hepatic parenchyma. It has not been fully investigated that the probability of the appropriate aortic or hepatic parenchymal enhancement at hepatic dynamic CT because conventional frequentism statistics cannot estimate these probabilities directly.^[9,10]^ Bayesian statistics has now permeated all the major areas of medical statistics, including clinical trials, epidemiology, meta-analyses and evidence synthesis, spatial modeling, longitudinal modeling, survival modeling, molecular genetics and decision-making in respect of new technologies because the Bayesian statistics can directly estimate probability distribution by applying the Bayesian probability theory.^[9,10]^ Additionally, the 120 kVp scan was widely used for hepatic dynamic CT; however, recent reports suggested that low kVp scan to increase the image contrast of hepatic dynamic CT by the photo-electric effect is more useful.^[11–14]^ The purpose of this study was to calculate ratio of the optimum contrast enhancement and optimal contrast material dose in the hepatic dynamic CT with difference kVp setting using the Bayesian probability theory.

## 2 Materials and methods

This retrospective study was approved by our institutional review board (No. E180723-2), with the requirement for informed patient consent being waived.

### 2.1 Patients

We included 2 retrospective cohorts in this study. First cohort includes 102 patients with liver cirrhosis who underwent a hepatic dynamic CT with 120 kVp between October 2014 and May 2015 at our institute. Second cohort includes 105 patients with liver cirrhosis who underwent a hepatic dynamic CT examination with 100 kVp between April 2016 and December 2016 at our institute. Their serum creatinine level was obtained within 3 months prior to contrast-enhanced examinations; their estimated glomerular filtration rate (eGFR) was calculated using the modified MDRD formula of the Japanese Society of Nephrology.^[11,15]^ From both groups we excluded patients with hemodialysis patients. Consequently, we excluded 7 patients with hemodialysis patients.^[16]^ Consequently, 100 patients were enrolled in the 120 kVp and 100 in the 100 kVp cohort; both cohorts were non-randomized. The patients’ sex, age, body height, and body weight at the time of examination was recorded (Table 1).

**Table 1:**
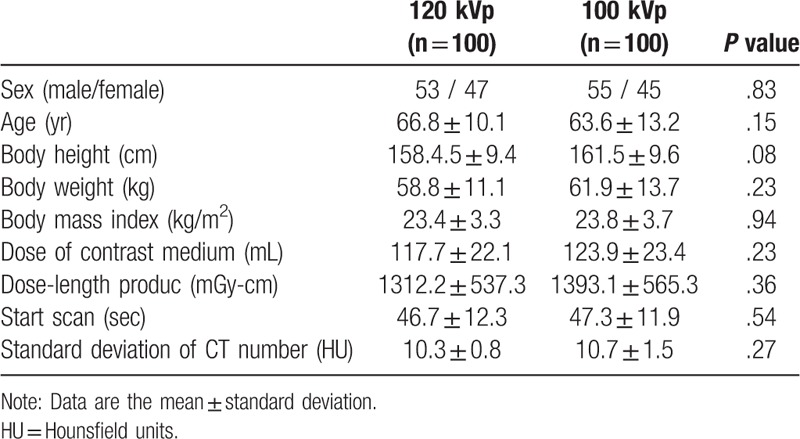
Patient demographics.

### 2.2 Scan protocol and image reconstruction

Contrast-enhanced multi-detector CT studies were performed on a 40-detector CT scanner (Lightspeed VCT; GE Healthcare, Milwaukee, WI) during a single breath-hold with the patient in the spine position. Scanning was from the top of the liver to the lower end of the kidney. The scanning parameters were 0.5-sec rotation, 5.0-mm detector row width, 0.516 helical pitch (beam pitch), 41.2-mm table movement, 50-cm scan field of view (FOV), 100 kVp or 120 kVp, and 100 to 770 mA using automatic tube current modulation (noise index 12). Depending on the geometry, the scanning time varied from 5 to 10 seconds.

We inserted a 20-gauge catheter into an antecubital vein and injected the contrast material (600 mgI/kg body weight, Omnipaque-300; Daiichi-Sankyo, Tokyo, Japan) with a power injector (Dual Shot; Nemoto-Kyorindo, Tokyo, Japan) in the course of 30 seconds. CT images of unenhanced-, hepatic arterial- and portal venous phase (HAP, PVP) scans were obtained; for the equilibrium phase we applied a 180-second delay. For HAP scanning we used a computer-assisted bolus tracking technique to synchronize the arrival of the contrast material at the abdominal aorta at the level of the celiac artery with the start of scanning. To monitor the arrival of the contrast material we performed axial scans of the abdominal aorta at the celiac artery level 20 second after the start of contrast injection. Scanning started automatically 15 second after contrast enhancement reached 100 HU in a region of interest (ROI) within the abdominal aorta. For PVP scanning we started the scan 20 second after the HAP.

### 2.3 Radiation dose and image noise measurements

Dose-length product (DLP) values displayed on the CT console were recorded. At each tube voltage we measured the image noise [standard deviation (SD) of the CT number] in the abdominal aorta at the celiac artery level on unenhanced scans. The image noise was recorded as the pixel value within an approximately 1.0-cm^2^ circular ROI.

### 2.4 Aortic and hepatic parenchyma attenuation measurements

In all patients we measured the CT number in the abdominal aorta at the celiac artery level on unenhanced scans and during HAP. We also measured the mean CT number in the hepatic parenchyma of the right- and left hepatic lobe at the celiac artery level on unenhanced and PVP scans. The CT number was recorded as the pixel value within an approximately 1.0-cm^2^ circular ROI. The degree of contrast enhancement was expressed as the change in the CT number calculated by subtracting the CT number on unenhanced- from the HU obtained on HAP and PVP images. We also calculated the rate of the optimal contrast enhancement (>280 HU in the ascending aorta and > 50 HU in the hepatic parenchyma) in each protocol.

### 2.5 Statistical analysis

For the comparison of the patient characteristics in interpatient variability under the 120 kVp protocols and under the 100 kVp protocols we used the Student *t* test. To compare the male/female ratio we used the x^2^ test. We compared the contrast enhancement in the abdominal aorta at HAP and hepatic parenchyma of the right- and left hepatic lobe at PVP between the two protocol groups.

Bayes’ theorem is stated mathematically as the following equation: 
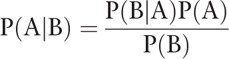


where A and B are events and P (B) ≠ 0.

P (A | B) is a conditional probability: the likelihood of event A occurring given that B is true. P (B | A) is also a conditional probability: the likelihood of event B occurring given that A is true. P (A) and P (B) are the probabilities of observing A and B independently of each other; this is known as the marginal probability.^[17]^

Using Bayesian analysis, the posterior probability distribution of the contrast enhancement and the contrast volume (mL/kg) for the optimal contrast enhancement (>280 HU in the ascending aorta and >50 HU in the hepatic parenchyma) in each protocol are stochastically sampled using Markov chain Monte Carlo (prior distribution - uniform distribution, five chains, 21,000 samples per chain and burn-in samples 1000: total 100,000 samples). We also estimate the probability of optimal contrast enhancement and the amount of contrast medium that offers optimal enhancement for 95% of the patients in each protocol. Differences of *P* < .05 indicated statistical significance. Statistical analyses were with free statistical software (version 3.0.2, the R project for statistical computing; http://www.r-project.org/).

## 3 Results

The mean DLP and SD values were 1312.2 ± 537.3 mGy-cm and 10.3 ± 0.8 HU at 120- and 1393.1 ± 565.3 mGy-cm and 10.7 ± 1.5 HU at 100 kVp. There was no significant difference in the radiation dose and image noise between the two protocols (*P* > .05, Table 1).

There were no statistically significant differences in the patients’ sex, age, body height, body weight, body mass index (BMI), and the contrast material dose (Table 1). Mean contrast enhancement and interpatient variabilities with respect to enhancement of the abdominal aorta during HAP and of the hepatic parenchyma of both hepatic lobes during PVP are shown in Table 2.

**Table 2:**
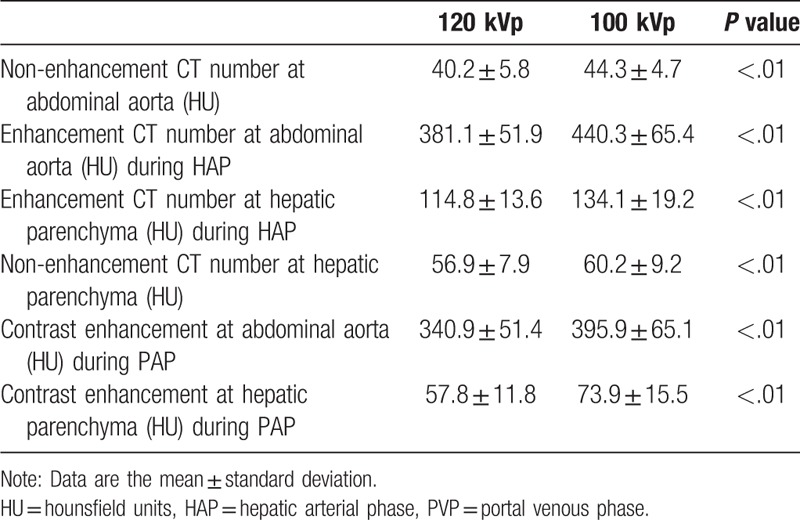
Mean CT number of the computed tomography (CT) attenuation.

Figure 1  shows the histogram of contrast enhancement. The mean contrast enhancement in the abdominal aorta was 340.9 ± 51.4 HU at 120- and 395.9 ± 65.1 HU at 100 kVp (*P* < .01); for the hepatic parenchyma these values were 57.8 ± 11.8 HU at 120- and 73.9 ± 15.5 HU at 100 kVp (*P* < .01). In the present study, 85.0% of measurements (85/100) demonstrated more than 280 HU at the abdominal aorta at 120 kVp, whereas 98.0% of measurements (98/100) demonstrated more than 280 HU at 100 kVp. For PVP, 77.0% of measurements (77/100) demonstrated more than 50 HU at the hepatic parenchyma at 120 kVp, whereas 94.0% of measurements (94/100) demonstrated more than 50 HU at 100 kVp.

**Figure 1:**
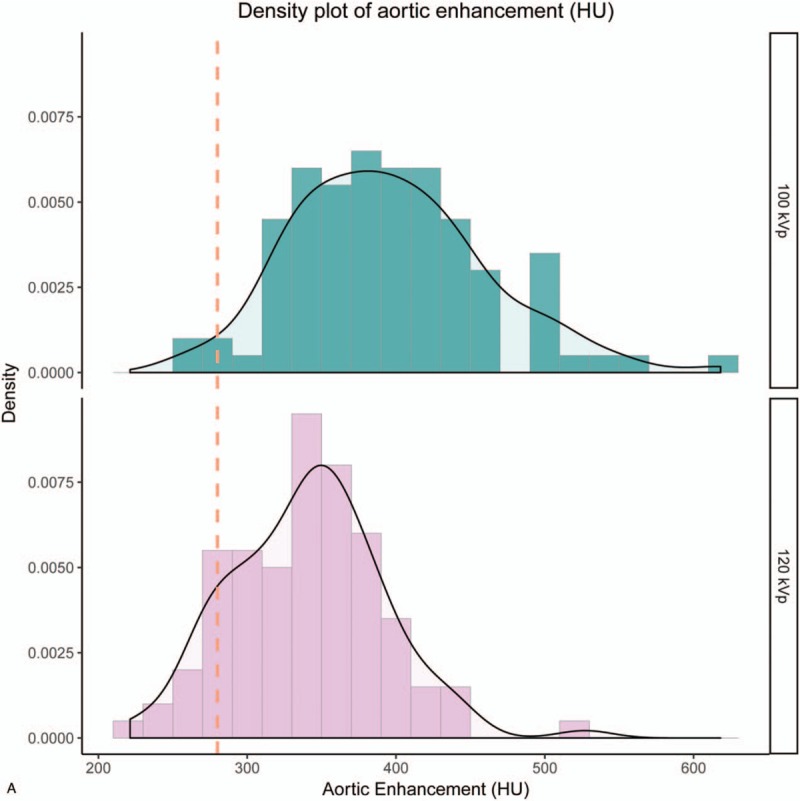
Histogram of contrast enhancement. The histogram of contrast enhancement in the abdominal aorta (A), and in the hepatic parenchyma (B). In the present study, 85.0% of measurements (85/100) demonstrated more than 280 HU at the abdominal aorta at 120 kVp, whereas 98.0% of measurements (98/100) demonstrated more than 280 HU at 100 kVp. For PVP, 77.0% of measurements (77/100) demonstrated more than 50 HU at the hepatic parenchyma at 120 kVp, whereas 94.0% of measurements (94/100) demonstrated more than 50 HU at 100 kVp.

**Figure 1 (Continued):**
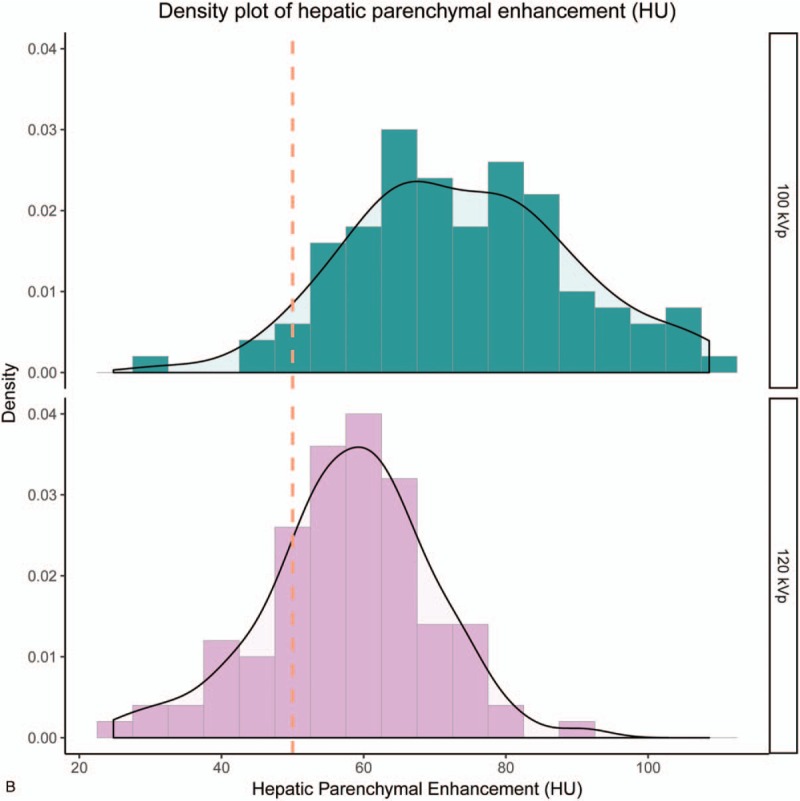
Histogram of contrast enhancement. The histogram of contrast enhancement in the abdominal aorta (A), and in the hepatic parenchyma (B). In the present study, 85.0% of measurements (85/100) demonstrated more than 280 HU at the abdominal aorta at 120 kVp, whereas 98.0% of measurements (98/100) demonstrated more than 280 HU at 100 kVp. For PVP, 77.0% of measurements (77/100) demonstrated more than 50 HU at the hepatic parenchyma at 120 kVp, whereas 94.0% of measurements (94/100) demonstrated more than 50 HU at 100 kVp.

**Figure 2:**
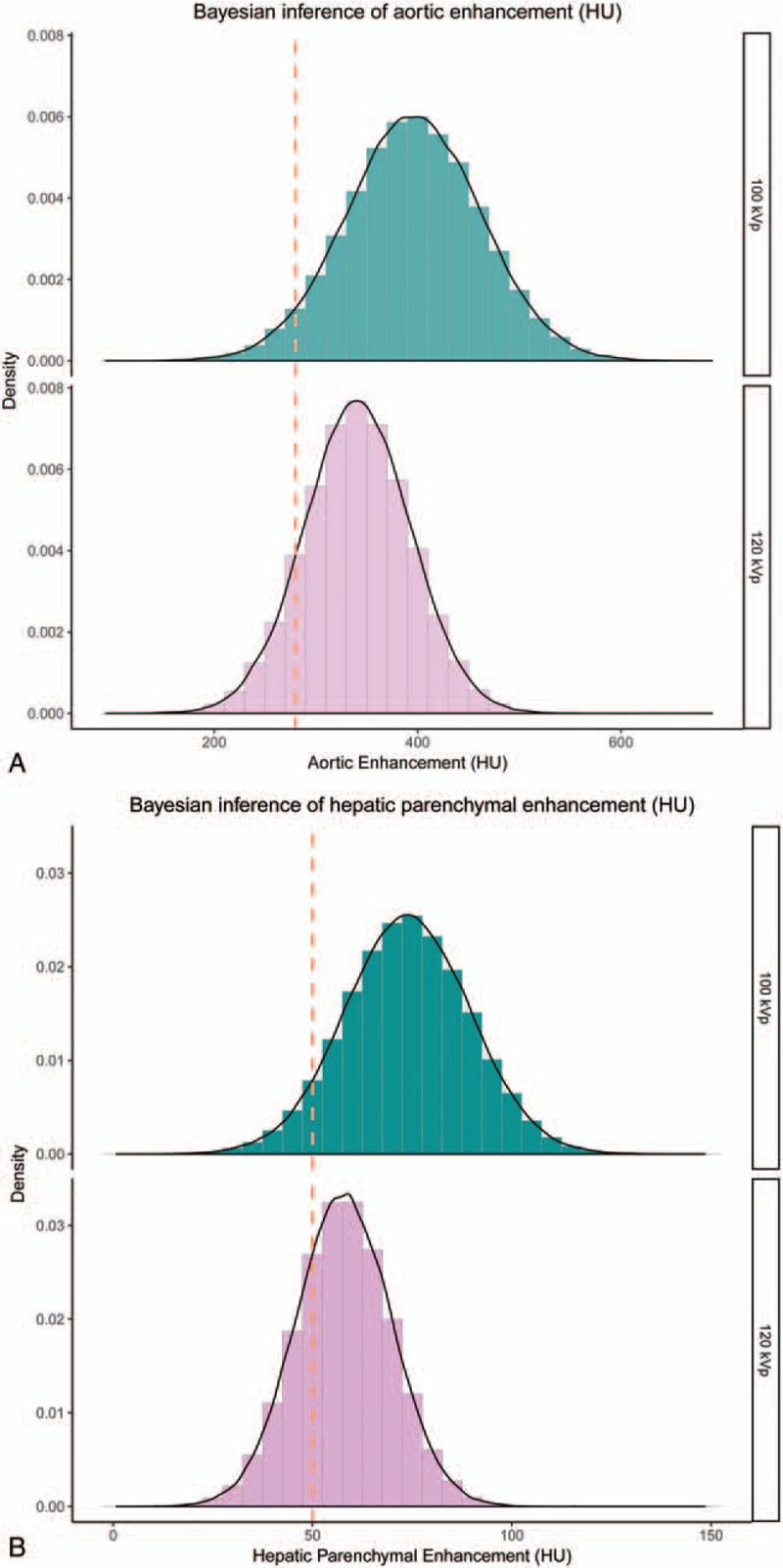
The posterior probability distribution by Bayesian inference of contrast enhancement. The posterior probability distribution for probability distribution of contrast enhancement in the abdominal aorta (A) and the hepatic parenchyma (B); the probability of achieving optimal contrast enhancement (>280 HU in the abdominal aorta); the probability of optimal enhancement for the abdominal aorta at HAP was 88.7 ± 2.5% at 120- and 98.8 ± 0.6% at 100 kVp; the probability of optimal enhancement for the hepatic parenchyma at PVP was 64.7 ± 3.8% at 120- and 95.3 ± 1.5% at 100 kVp.

Table 3 summarized the results of Bayesian inference for each protocol. Figure 2 shows the posterior probability distribution by Bayesian inference of contrast enhancement in the abdominal aorta and the hepatic parenchyma at 120- and 100 kVp. The probability of optimal enhancement for the abdominal aorta at HAP was 88.7 ± 2.5% at 120- and 98.8 ± 0.6% at 100 kVp. The probability of optimal enhancement for the hepatic parenchyma at PVP was 64.7 ± 3.8% at 120- and 95.3 ± 1.5% at 100 kVp.

**Table 3:**
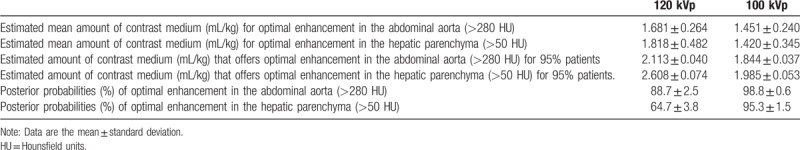
Bayesian inference between 100 kVp and 120 kVp protocol.

**Figure 3:**
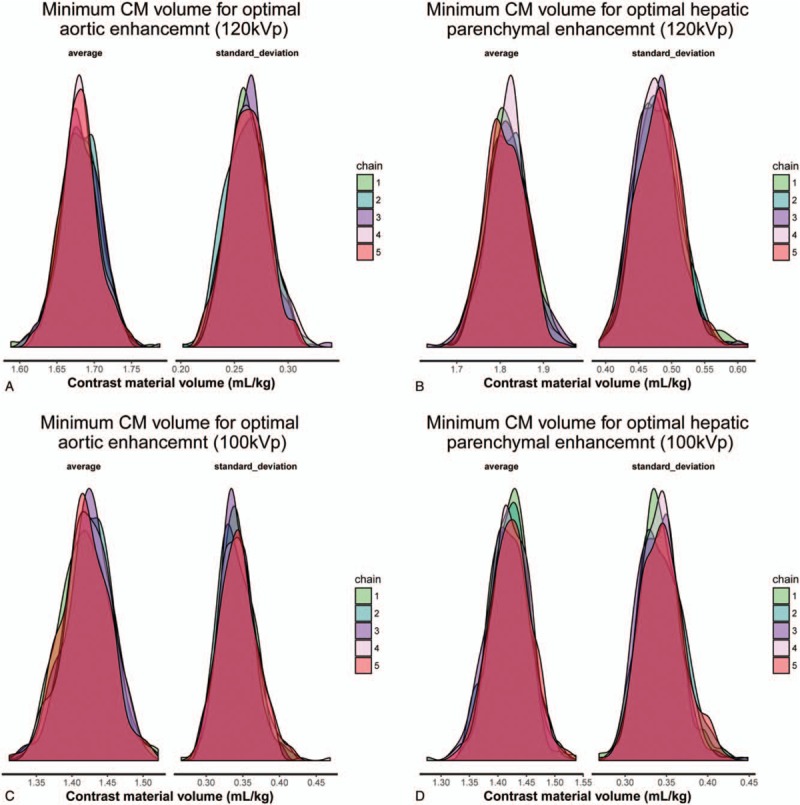
Posterior probability distribution of each Markov chain for the mean value and the standard deviation of the contrast medium volume (mL/kg) for optimal enhancement, a) 120 kVp – aortic enhancement, b) 100 kVp – aortic enhancement, (C) 120 kVp – hepatic parenchymal enhancement, and (D) 100 kVp – hepatic parenchymal enhancement. As a stochastic model describing a sequence of possible events in which the probability of each events depends only on state attained in the previous events show chain 1–5. The Bayesian inference is to find the parameters of the probability distributions. Usually the answers for the parameters are probability distributions themselves.

**Figure 4:**
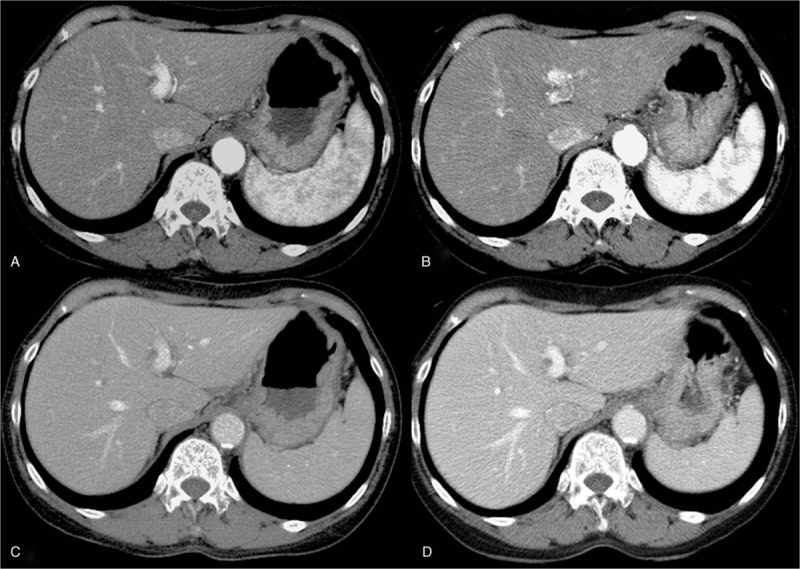
The patient was a 67-year-old woman with liver cirrhosis and her body height and weight were 164 cm and 54 kg. She happened to be included in both CT protocols. Axial images of (A) 120 kVp – hepatic arterial phase, (B) 100 kVp – hepatic arterial phase, (C) 120 kVp – portal venous phase, and (D) 100 kVp – portal venous phase are shown.

Figure 3 shows the posterior probability distribution of each Markov chain for the mean value and the standard deviation of the contrast medium volume (mL/kg) for optimal enhancement at 120- and 100 kVp. The estimated mean amount of contrast medium for optimal enhancement at HAP was 1.681 ± 0.264 mL/kg for 120 kVp scan, and 1.451 ± 0.240 mL/kg for 100 kVp scan. The estimated mean amount of contrast medium for optimal enhancement at PVP was 1.818 ± 0.482 mL/kg for 120 kVp scan, and 1.420 ± 0.345 mL/kg for 100 kVp scan. The estimated amount of contrast medium that offers optimal enhancement in HAP for 95% of the patients was 2.113 ± 0.040 mL/kg for 120 kVp scan, and 1.844 ± 0.037 mL/kg for 100 kVp scan. The estimated amount of contrast medium that offers optimal enhancement in PVP for 95% of the patients was 2.608 ± 0.074 mL/kg for 120 kVp scan, and 1.985 ± 0.053 mL/kg for 100 kVp scan. Figure 4 shows representative cases.

## 4 Discussion

Our results demonstrated that the probability of optimal hepatic parenchymal enhancement was not satisfactory when 120 kVp protocol was used at hepatic dynamic CT in thin adults. On the other hand, the100 kVp protocol yielded stable enhancement at hepatic dynamic CT.

It is generally well known that the enhancement by contrast material is much greater in lower kVp than in higher kVp; however, there is no previous report that evaluate ratio of optimal enhancement and optimal contrast dose in hepatic dynamic CT with different kVp settings. Our study suggested that the conventional frequentism statistics cannot estimate these probabilities directly; however, the Bayesian statistics can estimate the probability distributions of these parameters. We therefore believe the posterior probability offered by Bayesian statistics is suitable for practical use as compared with frequentist statistics. The original paper of Thomas Bayes establishing the Bayes theory is from just over 250years ago.^[18]^ The use of probability distributions to describe uncertain quantities distinguishes Bayesian statistics from frequentist statistics, which can lead to elegant solutions to many statistical problems.^[19,20]^

Our study suggested that the probability of adequate contrast enhancement was extremely improved by using the 100 kVp protocol in dynamic hepatic CT. Generally, lower kilovoltage image is vulnerable to make quantum noises, especially high BMI patients. Many researchers^[11–14]^ have studied that efforts to decrease kilovoltage have important implications in hepatic CT, noting that intrinsic tissue contrast rises with a decrease in kilovoltage, leading to superior ability to distinguish between two tissues with similar attenuation properties. In addition, because the K-edge of iodine is 33 keV, lower-tube voltage imaging lies closer to the iodine K-edge. In our results, by using 100 kVp protocol may obtain the sufficient contrast enhancement of abdominal aorta and parenchyma.

Although mean contrast enhancement was adequate at 120 kVp, on more than 30% of the images it was diagnostically insufficient. As calculation of the mean includes the lowest and highest values, in some patients, enhancement was inadequate. On the other hand, on more than 95% of 100 kVp CT images, contrast enhancement was sufficient, suggesting that the lower tube voltage technique is appropriate for hepatic dynamic CT studies.

Our results suggested that there are many variations in contrast enhancement at both protocols in hepatic CT. Even with the same injection protocol, among patients, there are variations in contrast enhancement because it is affected by the patient age, sex, body weight, height, BMI, body surface area, lean body weight (LBW), cardiovascular status, renal function, and the presence of other diseases.^[21]^ Awai et al especially^[22]^ reported that among body size parameters, LBW exhibited the strongest correlation with aortic and hepatic enhancement. Therefore, LBW tailor protocol may reduce the patient characteristics variations with arterial and hepatic enhancement.

This study however has other limitations. First, the range and mean BW of our patients was lower than that of North American and European individuals. Second, ours was a single center study and the study population was relatively small. Third, we did not exclude the effect of factors that might affect the enhancement such as liver function, cardiac output. Finally, we did not evaluate tumor to- liver contrast on images, vascular enhancement, and subjective image quality.

In conclusion, Bayesian inference suggested that the probability of optimal contrast enhancement at hepatic dynamic CT was 95.3% under 100 kVp; however, only 64.7% under the 120 kVp. The estimated amount of contrast medium that offers optimal enhancement for 95% of the patients was 2.608 ± 0.074 mL/kg for 120 kVp scan however, only 1.985 ± 0.053 mL/kg for 100 kVp scan.

## Author contributions

**Conceptualization:** Takanori Masuda, Takeshi Nakaura, Yoshinori Funama.

**Data curation:** Takanori Masuda, Takeshi Nakaura, Yoshinori Funama, Tomoyasu Sato, Yoriaki Matsumoto, Yukari Yamashita, Masao Kiguchi, Yasutaka Baba.

**Formal analysis:** Takeshi Nakaura, Tomoyasu Sato.

**Funding acquisition:** Takanori Masuda

**Investigation:** Takanori Masuda, Takeshi Nakaura, Toru Higaki, Yoriaki Matsumoto, Yukari Yamashita, Naoyuki Imada, Kazuo Awai.

**Methodology:** Takeshi Nakaura, Toru Higaki.

**Project administration:** Takanori Masuda

**Validation:** Takanori Masuda, Kazuo Awai.

**Visualization:** Tomoyasu Sato.

**Writing – original draft:** Takanori Masuda, Kazuo Awai.

**Writing – review & editing:** Takanori Masuda, Takeshi Nakaura, Yoshinori Funama, Yasuyuki Yamashita, Kazuo Awai.
